# Peer-supported self-management for people discharged from a mental health crisis team: a randomised controlled trial

**DOI:** 10.1016/S0140-6736(18)31470-3

**Published:** 2018-08-04

**Authors:** Sonia Johnson, Danielle Lamb, Louise Marston, David Osborn, Oliver Mason, Claire Henderson, Gareth Ambler, Alyssa Milton, Michael Davidson, Marina Christoforou, Sarah Sullivan, Rachael Hunter, David Hindle, Beth Paterson, Monica Leverton, Jonathan Piotrowski, Rebecca Forsyth, Liberty Mosse, Nicky Goater, Kathleen Kelly, Mel Lean, Stephen Pilling, Nicola Morant, Brynmor Lloyd-Evans

**Affiliations:** a Division of Psychiatry, University College London, London, UK; b Research Department of Primary Care and Population Health, University College London, London, UK; c Division of Psychology and Language Sciences, University College London, London, UK; d Department of Statistical Science, University College London, London, UK; e Camden and Islington NHS Foundation Trust, London, UK; f School of Psychology, University of Surrey, Guildford, UK; g Health Service and Population Research, King's College London, London, UK; h Brain and Mind Centre, University of Sydney, Sydney, Australia; i School of Social and Community Medicine, University of Bristol, Bristol, UK; j Avon and Wiltshire Mental Health Partnership NHS Trust, Bath, UK; k West London Mental Health Trust, London, UK; l Oxford Health NHS Foundation Trust, Oxford, UK

## Abstract

**Background:**

High resource expenditure on acute care is a challenge for mental health services aiming to focus on supporting recovery, and relapse after an acute crisis episode is common. Some evidence supports self-management interventions to prevent such relapses, but their effect on readmissions to acute care following a crisis is untested. We tested whether a self-management intervention facilitated by peer support workers could reduce rates of readmission to acute care for people discharged from crisis resolution teams, which provide intensive home treatment following a crisis.

**Methods:**

We did a randomised controlled superiority trial recruiting participants from six crisis resolution teams in England. Eligible participants had been on crisis resolution team caseloads for at least a week, and had capacity to give informed consent. Participants were randomly assigned to intervention and control groups by an unmasked data manager. Those collecting and analysing data were masked to allocation, but participants were not. Participants in the intervention group were offered up to ten sessions with a peer support worker who supported them in completing a personal recovery workbook, including formulation of personal recovery goals and crisis plans. The control group received the personal recovery workbook by post. The primary outcome was readmission to acute care within 1 year. This trial is registered with ISRCTN, number 01027104.

**Findings:**

221 participants were assigned to the intervention group versus 220 to the control group; primary outcome data were obtained for 218 versus 216. 64 (29%) of 218 participants in the intervention versus 83 (38%) of 216 in the control group were readmitted to acute care within 1 year (odds ratio 0·66, 95% CI 0·43–0·99; p=0·0438). 71 serious adverse events were identified in the trial (29 in the treatment group; 42 in the control group).

**Interpretation:**

Our findings suggest that peer-delivered self-management reduces readmission to acute care, although admission rates were lower than anticipated and confidence intervals were relatively wide. The complexity of the study intervention limits interpretability, but assessment is warranted of whether implementing this intervention in routine settings reduces acute care readmission.

**Funding:**

National Institute for Health Research.

## Introduction

Users of mental health services tend to be unenthusiastic about the prospect and the experience of receiving acute care, preferring interventions to help them recover, reintegrate with society, and achieve their personal goals.^1^ However, a large proportion of the scarce mental health resources in the UK and elsewhere are committed to inpatient and other acute care.^2^

In the National Health Service (NHS), crisis resolution teams are available nationwide as part of a strategy to reduce acute bed use.^3^, ^4^ Their target group is service users who are experiencing a crisis of sufficient severity for hospital admission to be considered. Clinicians in primary and secondary care refer service users whom they believe to meet this criterion, and in some catchment areas, self-referrals are also accepted. Guidance regarding the model requires that no hospital admission can occur without the agreement of crisis resolution teams. Some research evaluations have been positive, suggesting that crisis resolution teams can reduce inpatient admissions^5^, ^6^ and health-care costs,^7^ and increase service user satisfaction with acute care.^4^, ^7^ However, national implementation of the model has not resulted in a consistent reduction in bed use.^8^

A factor contributing to this failure to reduce admissions is a high rate of readmission to acute care,^9^ with more than half of users of crisis resolution teams readmitted within a year.^10^ A scoping review^11^ on interventions relevant to mental health crises found no robust evidence on how to prevent repeat crises in people leaving crisis care. Such evidence is needed in the UK and elsewhere to reduce heavy acute service use and support service users in making an uninterrupted recovery from crises.

**Research in context**

**Evidence before this study**

In 2013, we did a systematic review and meta-analysis on self-management interventions for people with severe mental illness. We searched Cochrane Central Register of Controlled Trials, CINAHL, DARE, Embase, Medline, and PsycINFO from their inception to June 30, 2013. Search terms for the interventions included “self-management”, “self-care”, “self-administration”, “self-evaluation”, “self-help”, “self-monitoring”, and “self-reinforcement”. We retrieved 35 papers meeting criteria for the review, of which 33 could be used in a meta-analysis. Most papers had very low Grading of Recommendations, Assessment, Development, and Evaluation ratings and short follow-up periods. Immediately after intervention, self-management programmes were more effective than controls for positive and negative symptoms of psychosis, psychological health symptoms, quality of life, hope, and self-rated and clinician-rated recovery; there were no significant differences between groups for service use outcomes, functioning, insight, or empowerment. Medium term (up to 12 months) pooled follow-up results showed effects on symptoms regressed to the mean in the year after treatment had ceased; however, quality of life, recovery, and hope remained significantly in favour of self-management. Studies did not focus on prevention of repeat crises in people using acute services. We also did a systematic review and meta-analysis of studies of peer-support interventions for people with severe mental health problems. 18 trials with considerable heterogeneity met criteria for this review, most again rated as low quality. There was little or no evidence that peer support was associated with positive effects on hospital admission, overall symptoms, or satisfaction with services. There was some evidence that peer support was associated with positive effects on measures of hope, recovery, and empowerment at and beyond the end of the intervention, although this was not consistent within or across different types of peer support. Thus, before our study, there was no substantial evidence on whether self-management interventions prevent readmission to crisis care services, and when we repeated our search in 2017 the same conclusion was drawn. Regarding peer support, evidence did not suggest effectiveness in reducing relapse or hospital admission among people with substantial mental health problems.

**Added value of this study**

We show an effect on readmission to acute care from a self-management intervention delivered by a peer support worker. This finding is novel, and of considerable potential importance because the intervention is feasible and acceptable, and service managers, planners, and users prioritise avoiding relapse and readmission to acute care.

**Implications of all the available evidence**

People discharged from community crisis services are often readmitted to acute care. This consumes resources that might otherwise be dedicated to longer term improvements in functioning and quality of life, prevents crisis services having intended effects on acute admissions, and impedes service users in pursuing their goals for their own recovery. Our trial shows the potential effectiveness of peer-delivered self-management in addressing this challenge, warranting investigation of the results of its implementation in routine settings. Self-management interventions are widely advocated and offer a straightforward mechanism for empowering service users and improving outcomes, but sustained and widespread implementation has not so far occurred. Our results also show that offering such an intervention during the period after a crisis is likely to be feasible and fruitful.

Self-management interventions have been developed in both mental and physical health care, and support people to actively manage their health problems.^12^ Interventions commonly include learning to anticipate and respond to signs of a crisis, and developing skills to manage symptoms and other difficulties. If successful, such interventions could reduce relapses and repeat acute care admissions following crises. In long-term conditions, self-management interventions are reportedly most successful when integrated with other care and as part of a service philosophy.^13^ Several modes of delivery can be used or combined in self-management interventions, including bibliotherapy or digital interventions, or involvement of clinicians in providing education and training.^14^ Involvement of peer workers with relevant personal experience is another potential mode of delivery. Peer workers could provide support and encouragement that is particularly warm and empathic because it is rooted in personal experience, and they provide service users with role models for recovery.^15^, ^16^ Thus, peer supporters appear particularly appropriate providers of interventions to promote self-management. North American trials of peer-supported self-management programmes such as the Wellness Recovery Action Plan^17^ and the Recovery Workbook^18^ report promising effects on symptoms and self-management skills. However, substantial evidence is not available regarding the effectiveness of these approaches, or of self-management interventions in general, in preventing relapse or acute care readmissions among people with mental health disorders.^19^

We tested whether an intervention to promote self-management in people leaving the care of mental health crisis teams reduced their subsequent rates of readmission to acute care. Peer support workers are increasingly employed in the NHS to support recovery, promoted by initiatives such as the NHS Confederation Implementing Recovery through Organisational Change project,^20^ but thus far the effectiveness of their efforts in reducing acute care readmission following a crisis has not, to our knowledge, been tested.

The primary hypothesis was that service users receiving the experimental intervention would be less likely to relapse (indicated by readmission to acute care) over 1 year than would those in the control group, who received treatment as usual enhanced by access to a self-management workbook. Secondary hypotheses were that being in the experimental rather than the control group would be associated with longer time to first readmission to acute care and fewer days in acute care over 1 year, and also with better self-rated recovery and illness management skills; greater satisfaction with services; fewer symptoms; less loneliness; and enhanced social networks at 4 month and 18 month follow-up interviews.

## Methods

### Study design and participants

The study was a rater-blinded, randomised controlled superiority trial done in six crisis resolution teams in England. Participants were identified from caseloads of crisis resolution teams, all aiming to operate according to the standard NHS model. All crisis resolution teams were contactable 24 h a day and saw service users mainly at home, offering short-term care during the crisis. Structured self-management interventions were not widely implemented in these teams' catchment areas.^21^

Participants were recruited after discharge by the crisis resolution teams. Eligible participants had been on the caseload for at least a week of one of the participating crisis resolution teams because of a crisis (including participants treated only by the crisis resolution team during the crisis episode and those initially admitted to hospital or a crisis house and then discharged with crisis resolution team support), had capacity and were willing to give written informed consent to participate, and consented to enter the trial within a month of discharge from the crisis resolution team. We excluded people who presented such a high risk to others that the crisis resolution team judged it unsafe for peer support workers to meet them even in a mental health service setting, those who were discharged to addresses outside the catchment area, and those who could not understand the intervention when delivered in English.

The published protocol gives greater detail of the methods.^22^ The trial was approved by the London Camden and Islington Research Ethics Committee (ref 12/LO/0988). A steering committee and a data monitoring committee oversaw the study.

### Randomisation and masking

Following baseline assessment, participants were randomly assigned with random permuted blocks into treatment and control groups at a ratio of 1:1, stratified by site. The treatment group received a peer-supported self-management intervention, based on a recovery workbook. Participants in the control group were sent the recovery book by post and received no other study intervention. Randomisation was done by either the study data officer or trial manager, generated by an online independent randomisation service.

Masking participants was not feasible. Participants and crisis resolution team staff were told allocation only after discharge from the crisis resolution team to ensure that allocation did not influence other plans for care. Data on readmission to acute care during the follow-up period was provided by administrators from participating NHS trusts, who were not informed of participants' treatment allocation, and entered in study databases by study research staff who were masked to treatment allocation. Research staff doing follow-up interviews at 4 months and 18 months were also not told participants' allocation and asked them not to disclose this at interview. Study statisticians analysing data were also masked to the allocation.

### Procedures

The peer-supported self-management intervention was adapted from recovery resources developed by Rachel Perkins, Julie Repper, and Miles Rinaldi, and their colleagues at South West London and St George's Mental Health NHS Trust.^23^ Selection and adaptation of the intervention is described in a companion paper.^24^ This process included literature searches and expert consultations to identify potential interventions; individual interviews with 41 service users exploring relevant views; stakeholder focus groups to inform adaptation of the intervention to a crisis resolution team context; and an uncontrolled feasibility study in which trained peer supporters delivered the intervention to 11 consenting participants.

Participants in the intervention group were offered ten individual sessions of 1 h each with a peer support worker. Sessions took place roughly once per week, aiming to conclude within 4 months. The peer support worker offered supportive listening and sought to instil hope through appropriate sharing of skills and coping strategies acquired in their own recovery. The intervention was structured around completion of a personal recovery workbook that included: setting personal recovery goals, making plans to re-establish community functioning and support networks after a crisis, using the recent crisis experience to identify early warning signs and formulate an action plan to avoid or attenuate relapse, and planning strategies to maintain wellbeing once a crisis had abated.

The workbook included boxes in which participants were encouraged to record observations, goals, and plans in each of these areas. Peer support workers were strongly encouraged to support participants in fully completing the workbook, but intervention time was sufficient for them also to spend time on more unstructured support and reflection on experiences and plans.

Peer support workers all had personal experience of using mental health services. Training included familiarising them with the workbook and how to support participants in using it, as well as more general content such as listening skills, cultural awareness, self-disclosure, and confidentiality. Group supervision was provided by clinicians from employing NHS trusts, typically once every 2 weeks, with additional support from the study team, including from an experienced peer support worker.

Participants in the control group were sent the personal recovery workbook by post, and were invited to complete it independently if they wished.

Peer support workers kept a brief anonymised log of the intervention, including sessions offered and attended and sections of the workbook completed. This log was shared with supervisors and the research team. Participants answered questions about their awareness and use of the workbook at interviews at 4 months.

Participants in both groups also received usual care, with no treatments withheld. A range of pathways were followed; participants were discharged to primary care if they did not need continuing specialist mental health care. Secondary mental health services in the trusts were configured in various ways, usually including community mental health teams as the main providers of continuing care, early intervention teams for psychosis, and assertive outreach teams.

Data were collected at baseline, in follow-up interviews at 4 months and 18 months, and from patient records. After written consent was provided and before allocation to groups, a study researcher collected baseline data from all participants in a structured interview. At 4 months and 18 months, researchers contacted participants to seek written informed consent for an interview. If obtained, a structured interview was held, including secondary outcome questionnaires. Data on acute care use were obtained from the data administrator of each trust by a blinded researcher.

After adjusting the intervention in response to findings from initial feasibility testing, a pilot randomised controlled trial was done in one trust (also included in the main trial) to test the feasibility and acceptability of trial procedures. 40 participants were recruited. It was agreed by the trial steering committee and funders that changes to study procedures and to the intervention following this internal pilot were sufficiently minimal for the internal pilot sample to be included within the main study sample. Data from the pilot trial were not analysed before proceeding to the main trial.

### Outcomes

The primary outcome was readmission of participants to an acute service (including acute inpatient wards, crisis resolution teams, crisis houses, and acute day care services) within 1 year after study entry. Secondary outcomes over 1 year of follow-up were days on the caseload of an acute service and time to first relapse (indicated by admission to an acute service). Secondary outcome measures assessed at 4 months and 18 months were: self-rated recovery, measured by total score on the Questionnaire on the Process of Recovery,^25^ a 22 item measure of self-rated recovery; self-management skills, rated by score on the patient version of the Illness Management and Recovery Scale,^26^ a 15 item measure of self-reported management of illness and functioning; client satisfaction, rated by total score on the Client Satisfaction Questionnaire,^27^ an eight item measure of respondents' overall satisfaction with mental health services; symptom severity, measured by the Brief Psychiatric Rating Scale,^28^ a 24 item scale of psychiatric symptoms rated by researchers on the basis of the participant's responses to a structured interview schedule; loneliness, as assessed by the University of California, Los Angeles (UCLA) Loneliness Scale,^29^ an eight item measure of self-rated loneliness; and social network, measured by the Lubben Social Network Scale,^30^ a six item measure of social contact with family and friends.

Further measures used to characterise the sample and to adjust in secondary analysis for variables known to be associated with the primary outcome included sociodemographic and clinical data (including age, sex, ethnicity, accommodation and living situation, employment status, educational attainment, and past service use, including admissions and compulsory admissions) and clinical diagnosis as recorded on electronic records using the International Classification of Diseases-10. Serious adverse events were actively monitored for both groups until completion of the 4-month follow-up interview.

Ethical approval was obtained for protocol amendment between the pilot and main trials to (a) add an interview to measure secondary outcomes 18 months after baseline and (b) add the UCLA Loneliness Scale and Lubben Social Network Scale to measures.

### Statistical analysis

We required a sample size of 440 to detect a difference in admission rates during the follow-up period of 50% in the control group versus 35% in the experimental group, with 80% power, 5% significance, and 1:1 allocation. This calculation allowed for clustering by peer support worker in the intervention arm only, assuming an intraclass correlation coefficient of 0·03.

We checked the assumptions underlying tests throughout. We did adjusted analyses if baseline characteristics were unbalanced. All analyses included only people for whom we had complete data available, using the groups to which the patients were randomised. We used Stata (version 14) throughout.

We compared readmission during the study period between randomisation groups using a logistic regression model with fixed effects for randomisation group, diagnosis (psychosis *vs* no psychosis), and NHS trust centre, and random intercepts to account for clustering by peer support worker. Participants in the control group were considered as individual clusters of size one.

For analysis of the secondary outcomes assessed by validated scales at 4 months and 18 months, we used linear regression with random intercepts (with peer support worker as the random effect), controlling for the baseline value of the outcome, condition (psychosis *vs* no psychosis), and centre. We had planned to use random effects Poisson regression to assess total days spent in acute care and a Cox regression frailty model for time to first readmission. However, on seeing the structure of the data, a zero inflated negative binomial with robust standard errors was more appropriate, given that more than half of participants had not spent any days in acute care since baseline. For time to first admission, the Cox regression frailty model did not converge, so we used a standard Cox regression with robust standard errors.

Few data were missing for the primary outcome, because it was derived from routinely recorded data. We quantified the extent of missingness for the other outcomes. We also investigated whether there were any patterns of missingness by creating dichotomous variables for each outcome to indicate whether that outcome was missing or not, and then investigating whether there were any associations between these variables and baseline characteristics using logistic regression models with random intercepts to account for clustering by peer support worker. We controlled for any baseline characteristics associated with missingness in sensitivity analyses to maintain the assumption of data missing at random.

This trial was registered with ISRCTN, number 01027104.

### Role of the funding source

The funder had no role in study design, data collection, data analysis, data interpretation, writing of the report, or the decision to submit. SJ, BL-E, GA, LMa, and RH had full access to the data. SJ made the final decision to submit for publication.

## Results

From 3288 mental health service users screened, 1848 were eligible and 441 participants were recruited (figure). 40 participants were recruited in the internal pilot between May 14, 2013, and Nov 12, 2013. The remaining 401 were recruited between March 12, 2014, and July 3, 2015. The final 18 month interview took place on Feb 23, 2017. 344 (78%) of 441 completed the 4-month follow-up interview, and 255 (58%) of 441 completed the 18-month interview. Most baseline characteristics were balanced between groups (table 1). The sample was diverse in terms of demographics, diagnosis, and service use history.

**Figure:**
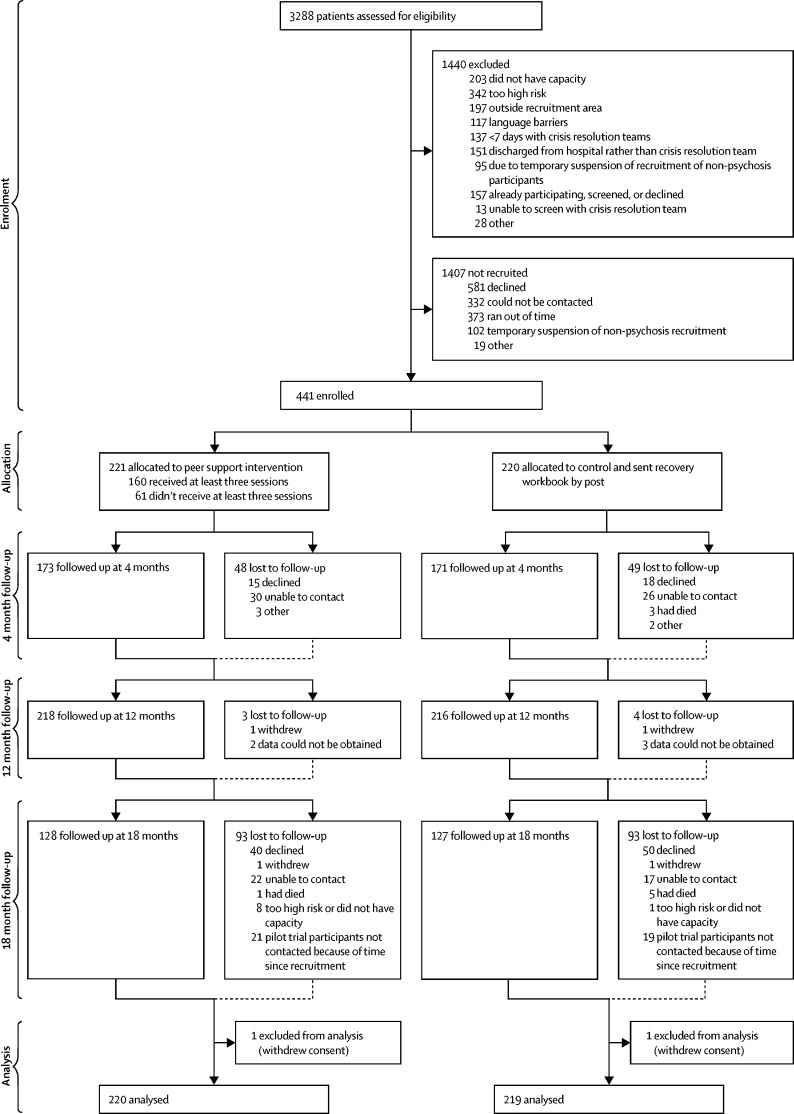
Study recruitment and retention

**Table 1:** Baseline characteristics

		**Intervention**	**Control**
Sex			
	Male	88/220 (40%)	87/218 (40%)
	Female	132/220 (60%)	131/218 (60%)
Age (years)		40 (13)	40 (12)
Ethnicity			
	White (UK and non-UK)	144/220 (65%)	141/218 (65%)
	Black (UK, African, Caribbean, and Other)	43/220 (20%)	41/218 (19%)
	Asian (UK, south Asian, Chinese, and Other)	14/220 (6%)	13/218 (6%)
	Other	19/220 (9%)	23/218 (11%)
	UK born	157/197 (80%)	147/196 (75%)
Marital status			
	Single	137/219 (63%)	146/219 (67%)
	Married or cohabiting	47/219 (21%)	49/219 (22%)
	Separated or divorced	28/219 (13%)	24/219 (11%)
	Widowed	7/219 (3%)	0/219 (0%)
Clinical diagnosis			
	Schizophrenia or schizoaffective disorder	28/213 (13%)	33/213 (15%)
	Bipolar affective disorder	27/213 (13%)	26/213 (12%)
	Other psychosis	12/213 (6%)	9/213 (4%)
	Depression	48/213 (23%)	53/213 (25%)
	Anxiety disorder	5/213 (2%)	2/213 (1%)
	Post-traumatic stress disorder	2/213 (1%)	5/213 (2%)
	Borderline or emotionally unstable personality disorder	16/213 (8%)	21/213 (10%)
	Other personality disorder	12/213 (6%)	6/213 (3%)
	Other or no clear diagnosis recorded	63/213 (30%)	58/213 (27%)
Lifetime admissions to psychiatric hospital			
	Never	69/199 (35%)	79/200 (40%)
	1	48/199 (24%)	38/200 (19%)
	2–5	48/199 (24%)	54/200 (27%)
	>5	34/199 (17%)	29/200 (15%)
Periods of support from a crisis resolution team			
	1	99/198 (50%)	93/200 (47%)
	2	39/198 (20%)	39/200 (20%)
	3–5	42/198 (21%)	44/200 (22%)
	6–10	11/198 (6%)	11/200 (6%)
	>10	7/198 (4%)	13/200 (7%)

Data are n/N (%) or mean (SD), where n is the number with the category in question and N is the total number of participants with data relating to the characteristic.

Readmission to acute care within 1 year was significantly lower in the intervention group than in the control group: 64 (29%) of 218 participants readmitted in intervention group versus 83 (38%) of 216 participants in the control group (odds ratio [OR] 0·66, 95% CI 0·43–0·99; p=0·0438; table 2). This difference persisted with planned adjustments (data not shown). Time to readmission was significantly longer in the intervention than in the control group (table 2). However, the number of days in acute care was not significantly different. Initial descriptive analyses showed an unexpected difference between groups in the number of days between randomisation and discharge from the index acute care admission (mean 8·6 days [SD 34·4] in the intervention group *vs* mean 2·9 days [SD 9·2] in the control group). In view of this, we did a post-hoc sensitivity analysis excluding these days and including only days that were part of acute care readmissions. In this analysis, participants in the intervention group had fewer days in acute care than did participants in the control group, but the difference was not significant (median 0 days [IQR 0–10] *vs* 0 days [IQR 0–22]; mean 13 days [SD 31] *vs* 19 days [SD 40]; 154 [71%] of 218 had no days in acute care *vs* 133 [62%] of 216; incident rate ratio [IRR] 0·90, 95% CI 0·66–1·23, p=0·5158). There was no evidence of a difference between groups in contact with the community mental health teams that were the main secondary care providers after discharge from a crisis resolution team (table 2).

**Table 2:** Outcomes

	**Intervention group**	**Control group**	**Association (95% CI)**	**p value**
**Primary outcome**				
Readmission to acute care over 1 year	64/218 (29%)	83/216 (38%)	OR 0·66 (0·43 to 0·99)*	0·0438
**Secondary outcomes**				
Satisfaction with mental health services at 4 months	26 (5)	24 (6)	DiM 1·96 (1·03 to 2·89)†	<0·0001
Satisfaction with mental health services at 18 months	26 (5)	25 (6)	DiM 0·98 (−0·50 to 2·46)†	0·1945
Days to first readmission to acute care during 1 year follow-up	112 (42 to 242)	86 (43 to 180)	HR 0·71 (0·52 to 0·97)	0·0291
Days spent in acute care during 1 year follow-up	0 (0 to 26)	0 (0 to 24)	IRR 1·01 (0·76 to 1·36)	0·9208
Self-management skills at 4 months	51 (8)	50 (8)	DiM 1·06 (−0·49 to 2·61)†	0·1807
Self-management skills at 18 months	53 (9)	52 (8)	DiM 1·24 (−0·77 to 3·26)†	0·2270
Self-rated recovery at 4 months	57 (16)	55 (16)	DiM 2·90 (0·08 to 5·72)†	0·0441
Self-rated recovery at 18 months	60 (13)	58 (70)	DiM 0·48 (−3·32 to 4·29)†	0·8032
Symptom severity at 4 months	39 (12)	41 (12)	DiM −1·08 (−3·17 to 1·01)†	0·3115
Symptom severity at 18 months	39 (12)	40 (13)	DiM −0·71 (−3·58 to 2·17)†	0·6306
Loneliness at 4 months	22 (3)	22 (4)	DiM 0·03 (−0·66 to 0·73)†	0·9254
Loneliness at 18 months	22 (4)	22 (4)	DiM −0·01 (−0·89 to 0·86)†	0·9805
Social network size at 4 months	12 (5)	12 (6)	DiM −0·06 (−1·02 to 0·90)†	0·9005
Social network size at 18 months	13 (6)	12 (6)	DiM 1·05 (−0·02 to 2·12)†	0·0549
**Service use**				
Community mental health team contacts at 12 months	7·00 (13)	7·60 (14)	DiM 0·16 (−2·28 to 2·61)†	0·8979

Data are n/N (%), mean (SD), or median (IQR). OR=odds ratio. DiM=difference in means. HR=hazard ratio. IRR=incident rate ratio.

* Adjusted for centre and condition.

† Adjusted for centre, condition, and baseline score.

At 4 months of follow-up, overall satisfaction with mental health-care received was greater in the intervention group than in the control group (table 2). There was also a significant difference in self-rated recovery favouring the intervention (table 2), but the difference was not significant in sensitivity analysis with adjustment for predictors of missingness (IRR 2·18, 95% CI −0·72 to 5·08). There were no other significant differences at 4 months. At 18 months, there was little evidence of any effect; the difference in social networks favoured the intervention but it was not statistically significant.

Regarding uptake of the intervention, 160 (72%) of 221 participants in the intervention group were treated according to the protocol—ie, they attended at least three meetings with their peer support worker, with a median of seven meetings (IQR 2–10) attended. 65 (33%) of 198 participants attended all ten meetings offered. Similar numbers of participants in each group reported that they had read the workbook at the 4 month interview (133 [84%] of 158 participants in the control group *vs* 142 [88%] of 162 in the intervention group). However, more participants in the intervention group reported using it to make written plans. Using the workbook to make written plans was reported by between 28% (38 of 138 participants for the section “Managing your ups and downs”) and 44% (61 of 138 participants for the section “Moving on after a crisis”) of participants in the control group, compared with between 58% (83 of 144 participants for the section “Your goals and dreams”) and 64% (92 of 144 participants for the section “Keeping well”) of the intervention group.

Three protocol breaches were reported to the study sponsors, trial steering committee, and data monitoring committee, resulting in eight experimental group participants not being offered the intervention per protocol. Two breaches were administrative errors by the data officer or peer support worker supervisor, resulting in participants not being offered the intervention by 4 months. One breach occurred when a peer support worker left after offering participants fewer than three sessions and was not replaced in time to offer the full intervention.

71 serious adverse events were identified (29 in the treatment group; 42 in the control group), including 55 readmissions to acute care, 11 attempted suicides, one participant charged with attempted murder, and four deaths (all in the control group: two suicides, two with unclear circumstances). All serious adverse events were assessed independently by the steering committee chair and the participant deaths and attempted murder by the data monitoring committee chair. None was judged related to the study.

## Discussion

The effect we observed for the primary outcome suggests that the peer-delivered self-management intervention reduces readmissions to acute care after a period of crisis team care, confirming the primary hypothesis, although the confidence intervals are relatively wide. If the finding that repeat periods of acute care were reduced by around a quarter is replicated in routine settings, the burden on the acute care system could be reduced substantially, and service users would have greater opportunities for sustained recovery. This trial adds promising evidence for self-management interventions for people with significant mental health problems: our systematic literature search did not find another substantial trial showing an effect on readmissions to acute care.^19^ It addresses the need for evidence on how to prevent repeat crises^11^ and provides the most robust evidence yet for the effectiveness of any peer-provided intervention in a UK secondary mental health setting.^31^ It also adds to evidence from US trials that structured, peer-delivered recovery-focused interventions including a relapse prevention component—such as a Wellness and Recovery Action Plan^18^ or Recovery Workbook^17^—can improve outcomes for mental health service users. A novel finding was support for the effectiveness of such an intervention to prevent relapse in users of crisis services.

We found that the intervention improved time to first acute readmission, but the number of days in acute care was not different, although in a post-hoc analysis including only days during readmissions to acute care, intervention participants were admitted for fewer days. However, no firm conclusions can be drawn from this analysis because of uncertainty around the treatment estimate, which is in part due to the high proportion of participants in each arm who were not readmitted.

Following planned adjustment for confounders, there was also a modest difference for overall satisfaction with care. The rating was of overall satisfaction with all care during and after crisis, so the impact of the trial intervention might have been diluted by views of other aspects of crisis management. Translating a positive effect on service users' views to routine care could help address recurrent findings of dissatisfaction with continuity of care and support following crises.^32^

For other secondary measures, there was a difference in perceived recovery at 4 months, but this was no longer significant in a sensitivity analysis adjusted for predictors of missingness. Effects on other 4 month outcomes, such as self-management skills and symptoms favoured the intervention, but the differences were not statistically significant. These differences had largely disappeared by 18 months, except for network size. These results were less positive than in some previous studies of self-management in mental health,^14^, ^17^, ^18^ and did not provide a robust basis for explaining the mechanism through which the effect on the primary outcome was achieved. Aspects of peer support or effects of formulating advance plans for crises that were not captured by our secondary measures might be relevant. Our secondary outcomes were global measures of recovery and illness management skills encompassing many aspects of participants' lives; more specific measures of potential mechanisms of action, such as confidence in recognising early warning signs, might be more informative. Of note, an Australian uncontrolled evaluation of peer support following an acute inpatient admission reported positive effects.^33^

With regard to strengths and limitations, the design and procedures were robust and followed international guidance, with oversight from a registered clinical trials unit. However, blinding of study participants was not feasible. The primary outcome was measured objectively and data obtained for 98% of participants. However, a limitation is that we could not clearly differentiate the effect on relapse during and following treatment. Regarding the primary outcome, readmissions were less frequent than anticipated when we powered the study. Although we have produced evidence that peer-delivered self-management reduces readmission to acute care, the confidence interval for the corresponding OR is relatively wide.

An acceptable response rate of nearly 80% was also achieved for the end-of-treatment secondary outcomes, although the response rate of just below 60% at 18 month follow-up is a limitation. Uptake of the intervention was fairly good, with 72% of the intervention group attending at least three meetings, although not all completed the workbook. Three meetings were considered by the research team and peer support workers to be sufficient to discuss and complete the personal recovery workbook, although the median of seven sessions attended will have allowed for more unstructured discussions with peer support workers.

Delivery of the intervention in a randomised trial had some effects on how it was implemented. About one in seven crisis resolution team service users screened and one in four of those eligible took part in the trial. However, we do not know how far this reflects the proportion of service users who might accept this intervention if offered as part of routine care. The intervention was designed to be broadly applicable, and an effect was obtained in a clinically and socially diverse group, suggesting potential for good generalisability. However, some clinical and social groups might benefit differently from the intervention.

We were not able to obtain anonymised data to directly compare our sample with those not recruited. However, a study^9^ using the Clinical Record Interactive Search tool for anonymising routine data examined an unselected sample of crisis resolution team users in two of six of the trusts in our study, allowing some simple comparisons. 48% of all users in one of these trusts and 45% in the other were male, compared with 40% in our sample; 68% and 50% were white compared with 65% in our sample; 14% and 20% were married compared with 21% in our sample; 24% and 35% had non-affective psychosis diagnoses compared with 19% in our sample; 29% and 37% had affective disorders compared with 36% in our sample. Thus, our sample was not highly unrepresentative of the crisis resolution team user population, although there may have been subtler differences, for example in motivation.

Our primary goal in designing the intervention was to maximise the likelihood that it would be effective. The result was a complex intervention with multiple components, including both peer support and self-management elements. Therefore, we cannot pinpoint the elements that resulted in its effectiveness. The control intervention, which involved sending participants the workbook through the post, resulted in a higher than anticipated proportion reading and making use of this tool. We do not know whether this intervention alone might be effective, and this active control might have diluted the impact of the experimental intervention. Most peer support workers were delivering an intervention of this type for the first time, and their confidence and familiarity with the intervention probably improved in the course of the trial.

This study should be replicated to confirm our findings. Better understanding of the critical components and mechanisms of effect of the intervention is also needed. Such understanding is particularly pertinent as improvements in symptoms and in social support are potential mechanisms for reduced rates of relapse, but differences on these outcomes were not significant. An evaluation of the CORE trial data will explore whether the relationship with the peer support worker and completion of the recovery plan have independent effects on outcomes, and will assess qualitatively experiences of the intervention. In addition, the intervention needs investigation from an implementation perspective. Research is needed on how to embed and sustain peer-supported self-management in routine services, and on associated outcomes and staff and service user experiences.

Our trial provides support for the wider and more systematic roll-out of practices that already attract considerable support from service planners and from service users themselves. Ideally, this implementation should be linked to mixed methods evaluation of the results of such roll-out in different contexts.
